# Frequency of significant virulence genes in gastric biopsies of *Helicobacter pylori*-positive patients with gastritis

**DOI:** 10.1186/s13568-023-01578-z

**Published:** 2023-07-06

**Authors:** Ebrahim Gholamhosseinzadeh, Hossein Ghalehnoei, Arash Kazemi Veisari, Somayeh Sheidaei, Hamid Reza Goli

**Affiliations:** 1 Immunogenetics Research Center, Faculty of Medicine, Mazandaran University of Medical Sciences, Sari, Iran; 2 Department of Medical Microbiology and Virology, Faculty of Medicine, Mazandaran University of Medical Sciences, Sari, Iran; 3 Department of Medical Biotechnology, School of Advanced Technologies in Medicine, Mazandaran University of Medical Sciences, Sari, Iran; 4 Gut and Liver Research Center, Non-Communicable Disease Institute, Mazandaran University of Medical Sciences, Sari, Iran; 5 Department of Pathology, Faculty of Medicine, Mazandaran University of Medical Sciences, Sari, Iran

**Keywords:** *Helicobacter pylori*, *CagA*, *VacA*, *DupA*, *IceA1&2*, *OipA*

## Abstract

*Helicobacter pylori* is one of the most common bacteria affecting human societies worldwide, and is mainly associated with gastrointestinal complications due to different virulence factors. This study aimed to investigate some virulence genes of *H. pylori* in gastric biopsies of patients with gastritis in Sari city, North of Iran. Informed consent forms were obtained from the studied patients, and those who needed endoscopy were included in the study. To evaluate the prevalence of c*agA*, *iceA1*, *iceA2, vacA*, *dupA*, and o*ipA* genes, gastric biopsies with positive or negative rapid urease test were collected from 50 patients (25 in each group) with gastro-duodenal diseases. The bacterial DNAs were extracted by a specific kit, and the presence of the genes was analyzed by PCR using specific primers. Eighteen (72%) biopsies from 25 *H. pylori*-positive samples were c*agA*-positive, while 17 (68%) biopsies contained the *vacA* gene, and 11 (44%) samples had both *vacA* and *cagA* genes. However, 16 (64%), 12 (48%), 13 (52%), and 14 (56%) biopsies contained *dupA*, *iceA1*, *iceA2*, and *oipA* genes, respectively. Due to the significant role of the studied virulence factors in the pathogenicity of *H. pylori*, the high prevalence of these factors in biopsies of patients with gastritis is a concern needing to the management in this region.

## Introduction

*Helicobacter pylori* is one the most prominent bacteria causing gastrointestinal diseases, especially in developing countries. This class I carcinogenic organism causes chronic gastritis, gastric ulcers, and cancers (Suerbaum and Michetti 2002). Chronic gastritis can resulte from immune responses of gastric mucosa against *H. pylori* infection in humans (Wroblewski et al. 2010). Some virulence factors are involved in pathogenesis of this organism. Some outer membrane proteins, including sabA, oipA, babA, babB, and iceA1 along with cagA and vacA cytotoxines, peptidoglycan, adhesins, and some enzymes, such as mucinase and urease, are the most significant in *H. pylori* (Wroblewski et al. 2010). The cagA protein can cause mucosa-associated lymphoid tissue (MALT) B-cell lymphoma and gastric adenocarcinoma related to the *H. pylori* infection after tyrosine phosphorylation by Src family kinases (Matos et al. 2013). The vacA cytotoxine can cause a strong vacuolation and directly disruption of the mitochondrial function of the gastric cells (Kumar et al. 2008).

In additin, the iceA protein that can overexpress by interaction between *H. pylori* and human epithelial cells, was more detected in patients with peptic ulcer and gastritis (Abu-Taleb et al. 2018). The presence of iceA can cause increased expression of gastric mucosal interleukin 8 (IL-8) and the emergence of acute inflammation (Abu-Taleb et al. 2018). The iceA is a significant *H. pylori* virulence factor, and the bacterial adhesion to gastric epithelial cells can stimulate the *iceA1* gene transcription in vitro (Howden and Hunt 1998). The dupA stimulates the in vivo and in vitro production of IL-8 and IL-12 from the gastric mucosa and epithelial cells, respectively. This virulence factor may cause duodenal ulcers or reduce gastric cancers in some populations (Lu et al. 2005). Besides, the oipA protein can induce the gastric inflammation and actin dynamics by phosphorylation of several signaling pathways, including the *cagPAI* (cag pathogenicity island) (Dossumbekova et al. 2006), indicating a potential interaction between the oipA and cagA (Horridge et al. 2017). However, additional studies are needed to explain the precise role of oipA in *cagPAI* pathway (Horridge et al. 2017). Due to the significant role of these virulence factors in the pathogenicity of *H. pylori*, this study aimed to investigate the frequency of *cagA, vacA, dupA, iceA1 & 2*, and *oipA* genes in gastric biopsies of *H. pylori*-positive patients with gastritis in Sari city, north of Iran.

## Materials and methods

### Patients, samples, and ethical approval statements

In this case–control study, bacterial strains were isolated from patients with stomach diseases referred to the endoscopy department of Baghban Medical Center affiliated to Mazandaran University of Medical Sciences. Twenty-five participants in the case group were *H. pylori*-positive and 25 patients in the control group were *H. pylori*-negative. According to the results of previous studies and using the sample size formula, and considering the maximum error of 0.05 points, the confidence level of 0.95, and the ratio of 0.97, 50 samples were estimated. This study was directed in agreement with the Declaration of Helsinki. A written informed consent form was delivered by the patients or a close relative before sampling. Classifying information of each specimen was kept secret. We received the clinical samples of patients without names from the endoscopy center and performed the desired tests on these samples. In addition, the Iran National Committee for Ethics in Biomedical Research approved this study with the national ethical code IR.MAZUMS.REC.1397.382. In this study, we obtained corporal and antral gastric biopsies for the histopathological evaluation and two additional samples from antrum for the rapid urease test and molecular detection in microbiology laboratory. However, to confirm the role of *H. pylori* in a patient’s infection, we used the stool-antigen-detection test in addition to histopathological examination and RUT.

### Rapid urease test (RUT) and stool antigen detection

*Helicobacter pylori* converts urea to ammonia and carbon dioxide due to its urease activity. Ammonia changes the color of the urea broth culture media (Merck, Germany) in the presence of phenol red by changing the pH. An Eppendorf tube containing 1 ml of a 10% urea broth was used to perform this test. A biopsy was placed into this medium and the positive result was detected by an orange to pink change color of the medium within 1–3 min (Kumar et al. 2008). Also, the *H. pylori* antingen detection in stool sample was used as a simple and non-invasive option for diagnosis (Calik et al. 2016). The rapid test strip (Mascia Brunelli, Italy), was used to detect the *H. pylori* antigen, according to the manufacturer’s instructions. Brifely, 150 mg of the stool sample put into the tube containing 0.5 ml buffer and shaked it to dispersion. Then, one drop of the suspension was added to the test strip and the positive result was detected after 10 min.

### Histopathological staining

The histopathologic staining, as a standard method, can use to directly observing the *H. pylori* in gastric biopsies. The gastritis severity was assessed using updated Sydney system on hematoxylin and eosin staining. Biopsy specimens were obtained from two sites (corpus and antrum), and evaluated for neutrophil activity, intestinal metaplasia, atrophy, mononuclear cells, and *H. pylori* density. The scores were graded on a scale of 0–3, including absent inflammation (Grade 0), mild inflammation (Grade 1), moderate inflammation (Grade 2), and severe inflammation (Grade 3) (Dixon et al. 1996).

Although hematoxylin and eosin (H&E) staining is a routine technique in daily detection (Molaei et al. 2010), our pathologist used the Giemsa staining to detect *H. pylori* due to its high sensitivity (Koehler et al. 2003). First, the slides were placed in xylene 1, 2, and 3 (each for 2 min). Then, the slides were located two times in absolute alcohol (each for 2 min), and ten times dipped in each 96 and 70% alcohol. After rinsing with distilled water, the slides put in a Giemsa solution overnight. Then, the pathologist rinsed the slides with distilled water and 0.5% acetic acid. Finally, the slides washed with running water and then air-dried.

### DNA extraction and polymerase chain reaction (PCR)

Genomic DNAs of the bacteria were extracted from the biopsies using a DNA extraction kit (SinaClon, Iran), according to the manufacturer’s instructions. After DNA extraction, the specific primer pairs shown in Table 1 were used to detect the *cagA*, *vacA*, *iceA1*, *iceA2*, *dupA*, and *oipA* genes using the PCR technique. The PCR was done in a 15 μl final volume containing 7.5 μl of Master Mix (Ampliqon, Denmark), 300 ng of the extracted DNA, ten pmol of each primer, and distilled water up to 15 μl. The PCR conditions were included one cycle of primary denaturation at 95 °C for 5 min followed by 34 cycles, including denaturation at 95 °C for 30 s, primer annealing at different temperatures shown in Table 1 for 45 s, and the extension step at 72 °C for 30 s. In addition, the final extension step was performed for 10 min at 72 °C. The electrophoresis of PCR products was done on a 1% agarose gel (Sigma, Germany) containing safe stain (SinaClon), and the results were observed by a gel documentation (UVITEC Cambridge, UK).

**Table 1:** Sequences of the primer paires used in this research

Target genes	Primers sequences (5ʹ to 3ʹ)	Anealing temperatures (°C)	Products length (bp)	References
*cagA*	F-GATAACAGGCAAGCTTTTGA-3′ R-CTGCAAAAGATTGTTTGGCA-3′	50	349	This study
*vacA*	F-CAATCTGTCCAATCAAGCGAG-3′ R-GCGTCAAAATAATTCCAAGG-3′	58	567	This study
*dupA*	F-ACGATTGAGCGATGGGAATA-3′ R-AAGCTGAAGCRTTTGTAACGA-3′	53	467	This study
*iceA1*	F-GTGTTTTTAACCAAAGTATC-3′ R-CTATAGCCASTYTCTTTGCA-3′	50	247	This study
*iceA2*	F-GTTGGGTATATCACAATTTAT-3′ R-TTRCCCTATTTTCTAGTAGGT-3′	50	235	This study
*oipA*	F-GCTATATTMAAGGACAAGGYAG-3′ R-CCAGGAA*CAGA* ACCAACR-3′	53	316	This study

bp; base pair

### Statistical analysis

The Statistical Package for the Social Sciences (SPSS) software version 22 was used for analyzing the data and a data comparison performed by the Chi-square test. *P-values* < 0.05 were considered as statistical significant.

## Results

This study was performed on 50 gastric biopsies taken by a gastroenterologist from patients with gastritis. The rapid urease test, stool antigen detection, and histopathological staining were used to detect *H. pylori* in biopsies. However, 25 specimens were grouped as *H. pylori-positive*, and others belonged to the *H. pylori-*negative group. According to the histopathological staining, patients were grouped as chronic active gastritis, severe active gastritis, and normal-looking mucusa.

**Fig. 1:**
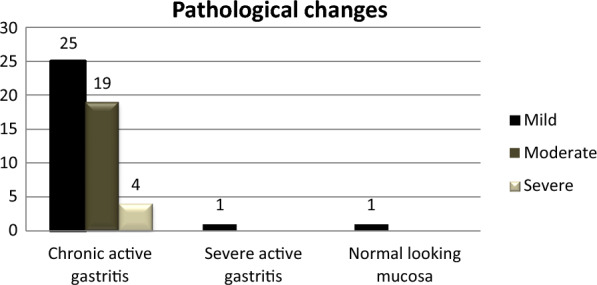
The pathological changes detected in gastric biopsies collected in this study

Out of 50 samples taken from patients, 48 (96%) exhibited a clinical presentation of chronic active gastritis. Among these 48 patients, 19 (39.58%) had a chronic moderate gastritis, 25 (52.08%) participants showed a chronic mild gastritis, and the remaining 4 (8.33%) patients exhibited a chronic active severe gastritis (Fig. 1). On the other hand, only one sample showed a view of severe active gastritis, and one of them had a normal-looking mucosa. Also, we detected that 12 (24%) patients had an intestinal metaplasia as precancerous lesions, while two patients in this group were *H. pylori*-negative. However, among 25 *H. pylori*-positive patients, 10 (40%) showed intestinal metaplasia, from which 2 (20%) patients had a sever metaplasia, while 6 (60%) had a moderate metaplasia, and 2 (20%) others showed a mild metaplasia.

**Fig. 2:**
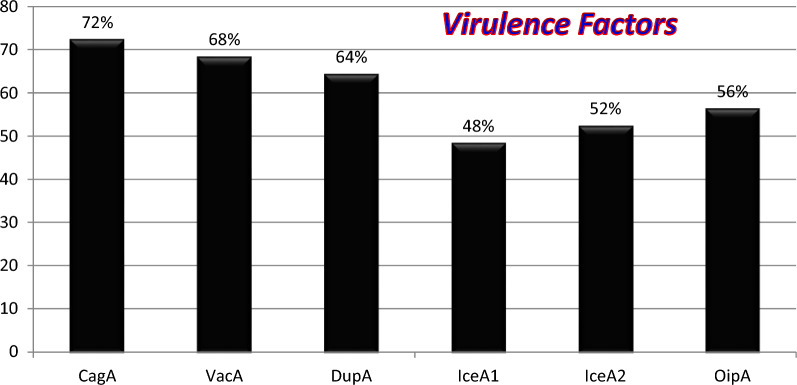
Prevalence of *H. pylori* virulence genes in biopsies collected in this study

According to the PCR test among 25 *H. pylori*-positive specimens, 18 (72%), 17 (68%), 16 (64%), 12 (48%), 13 (52%), and 14 (56%) specimens were contained *cagA*, *vacA*, *dupA*, *iceA1*, *iceA2*, and *oipA*, respectively (Fig. 2). In addition, 11 (44%) samples were positive for both *cagA* and *vacA* genes. Also, the complete information about our specimens is shown in Table 2. According to this table, we detected some specimens with negative results for *H. pylori* which contained some virulence genes.

**Table 2:** The complete information of the samples along with the PCR results in the present study

ROW	RUT	Stoll antigen detection	Pathological outcomes	*CagA*	*VacA*	*OipA*	*IceA1*	*IceA2*	*DupA*
1	−	+	Active chronic gastritis, moderate intestinal metaplasia, *H. pylori* = positive	+	+	1	−	+	+
2	−	−	Chronic gastritis, mild intestinal metaplasia, *H. pylori* = negative	+	−	+	+	+	−
3	−	+	Focal active chronic gastritis, mild intestinal metaplasia, *H. pylori* = positive	−	+	+	−	−	+
4	+	+	Active chronic gastritis, moderate to severe, *H. pylori* = positive	−	+	−	−	−	+
5	−	−	Chronic gastritis, mild, *H. pylori* = negative	−	−	+	−	+	+
6	+	+	Active chronic gastritis, moderate, *H. pylori* = positive	+	−	+	+	−	−
7	+	+	Active chronic gastritis, moderate, *H. pylori* = positive	−	−	+	+	−	−
8	−	−	Normal looking mucosa, *H. pylori* = negative	−	−	−	+	−	−
9	−	−	Chronic gastritis, moderate, *H. pylori* = negative	+	−	+	+	−	+
10	+	+	Active chronic gastritis, moderate intestinal metaplasia, *H. pylori* = positive	+	+	+	+	+	+
11	−	−	Chronic gastritis, moderate, *H. pylori* = negative	+	−	−	−	−	+
12	+	+	Active chronic gastritis, severe intestinal metaplasia, *H. pylori* = positive	+	+	−	−	+	−
13	−	−	Active chronic gastritis, mild, *H. pylori* = negative	−	−	−	−	−	−
14	−	−	Active chronic gastritis, moderate, *H. pylori* = positive	−	−	−	−	−	+
15	−	−	Chronic gastritis, mild, *H. pylori* = negative	+	−	+	+	+	+
16	−	+	Active chronic gastritis, moderate intestinal metaplasia, *H. pylori* = positive	+	+	+	−	+	+
17	−	+	Active chronic gastritis, moderate intestinal metaplasia, *H. pylori* = positive	+	+	+	−	−	+
18	+	+	Active chronic gastritis, severe, *H. pylori* = positive (severe)	−	−	−	+	+	−
19	+	+	Active chronic gastritis, moderate, *H. pylori* = positive	+	+	+	+	+	+
20	−	−	Chronic gastritis, mild, *H. pylori* = negative	+	+	+	+	−	+
21	−	+	Active chronic gastritis, mild, *H. pylori* = positive	+	−	+	−	+	−
22	+	+	Active chronic gastritis, moderate intestinal metaplasia, *H. pylori* = positive	−	+	+	+	+	+
23	+	+	Active chronic gastritis, moderate, *H. pylori* = positive	−	+	+	−	−	+
24	+	+	Active chronic gastritis, moderate, *H. pylori* = positive	−	+	+	−	−	+
25	−	−	Chronic gastritis, mild, *H. pylori* = negative	−	−	−	−	−	−
26	−	+	Active chronic gastritis, moderate intestinal metaplasia, *H. pylori* = positive	+	−	−	−	−	+
27	+	+	Active chronic gastritis, moderate, *H. pylori* = positive	+	+	−	+	+	−
28	+	+	Active chronic gastritis, mild, *H. pylori* = positive	−	+	−	−	+	+
29	+	+	Active chronic gastritis, mild intestinal metaplasia, *H. pylori* = positive	+	−	−	−	+	+
30	−	−	Chronic gastritis, mild, *H. pylori* = negative	−	+	+	+	−	−
31	−	−	Chronic gastritis, mild, *H. pylori* = negative	−	−	+	+	−	+
32	−	−	Chronic gastritis, moderate intestinal metaplasia, *H. pylori* = negative	−	−	−	+	−	+
33	−	−	Chronic gastritis, moderate, *H. pylori* = negative	−	+	+	−	+	+
34	+	+	Active chronic gastritis, severe, *H. pylori* = positive	+	+	+	+	−	−
35	−	−	Chronic gastritis, mild, *H. pylori* = negative	−	+	+	−	+	−
36	−	−	Chronic gastritis, moderate, *H. pylori* = negative	−	−	−	−	−	−
37	+	+	Active chronic gastritis, severe intestinal metaplasia, *H. pylori* = positive	+	+	+	−	+	+
38	+	+	Active chronic gastritis, moderate, *H. pylori* = positive	+	+	+	+	−	+
39	+	+	Active chronic gastritis, mild, *H. pylori* = positive	+	−	−	−	−	+
40	+	+	Active chronic gastritis, mild, *H. pylori* = positive	+	+	−	−	+	−
41	−	−	Chronic gastritis, mild, *H. pylori* = negative	−	−	+	+	+	−
42	−	−	Chronic gastritis, mild, *H. pylori* = negative	−	−	−	+	−	+
43	−	−	Chronic gastritis, mild, *H. pylori* = negative	+	+	−	+	+	+
44	−	−	Chronic gastritis, mild, *H. pylori* = negative	+	−	+	−	+	+
45	−	−	Chronic gastritis, mild, *H. pylori* = negative	−	−	−	−	−	−
46	−	−	Chronic gastritis, mild, *H. pylori* = negative	−	−	−	−	−	−
47	−	−	Chronic gastritis, mild, *H. pylori* = negative	−	−	−	−	−	−
48	−	−	Chronic gastritis, mild, *H. pylori* = negative	−	−	−	−	−	−
49	−	−	Chronic gastritis, mild, *H. pylori* = negative	−	−	−	−	−	−
50	−	−	Chronic gastritis, mild, *H. pylori* = negative	−	−	−	−	−	−

## Discussion

*Helicobacter pylori* is one the most common causes of gastrointestinal diseases, such as gastritis, gastric or duodenal ulcers, and rarely gastric lymphoma or cancer (Feng et al. 2013). According to the results of the present study, out of 50 biopsy specimens, 41 (82%) were positive for the presence of different virulence genes of *H. pylori*. However, the diagnostic methods used in this study indicated 25 (50%) *H. pylori-positive* specimens. This discrepancy may be due to the existence of small amounts of bacterial DNA in healthy individuals that can detect by molecular testing. In addition, these strains may even carry virulence factors. However, seven RUT-negative samples of the present study were pathologically positive for *H. pylori*, which may be due to the relative quality of the urease kit or insufficient accuracy in performing the pathological tests.

It has been shown that the prevalence of *H. pylori* and chronic active gastritis is higher and increasing at younger ages, while decreases after the fifth decade (Homan et al. 2009). In addition, the incidence of chronic gastritis and intestinal metaplasia is lower at Youngers and increases gradually. On the other hand, the percentage of negative cases of *H. pylori* in chronic gastritis and intestinal metaplasia is higher than its positive cases (Homan et al. 2009). However, in the present study, 48 (96%) samples exhibited chronic active gastritis, while just one specimen was detected as severe active gastritis, and one another showed a mucus with a normal appearance.

Molecular analysis of the present study showed that the highest frequency of virulence genes was related to the *cagA* (72%) gene, from which most of them had chronic gastritis (11 cases of moderate, 9 cases of mild, and 3 cases of severe). In addition, the lowest frequency (48%) was related to the *iceA1* gene. However, Molaei et al. (2010) reported a 79.6% prevalence of the *cagA* gene which was almost consistent with the results of our study, indicating the major role of *cagA* in primary gastritis. Moreover, Rasi-Bonab et al. (2021) in Tabriz reported the frequency of the *cagA* gene as 63%. On the other hand, we found that seven *H. pylori*-negative biopsies in our study were contained the *cagA* gene, which may be due to a past infection. Also, Vannarath et al. (2014) were studied 119 *H. pylori*-infected patients with dyspepsia and found that 83 (69.7%), 13 (10.9%), 20 (16.8%), and 3 (2.5%) patients had acute gastritis, chronic gastritis, duodenal ulcers, and gastric cancer, respectively. However, the *cagA* gene was presented in 99.2% of their biopsies (Vannarath et al. 2014). Besides, another study conducted by Palframan et al. (2012) showed that the vacA is a key toxin in gastric epithelial cell damage and the emergence of gastric ulcers. It can be concluded that this factor can cause more bacterial colonization, tissue inflammation, and stronger immune response in gastric tissue.

Moreover, Koehler et al. examined the *H. pylori* genomes isolated from 92 paraffin tissue samples from patients with gastric adenocarcinoma and MALT B-cell lymphoma, and found that the prevalence of *iceA1* and *iceA2* genes was 6.5 times higher in patients with adenocarcinoma (Koehler et al. 2003). In our study, 20 (40%) specimens were *iceA1*-positive, while 12 of them were *H. pylori-positive* according to the pathological staining. Also, we found that 21 (42%) samples were *iceA2*-positive, while 14 of them were *H. pylori-positive*. These discrepancies may be because the person was actually *H. pylori-positive*, but had a past treated infection by remained DNAs of the bacteria. Another study conducted in Turkey (neighboring Iran) by Mustafa Akar et al. (2022) showed that 69.6% of their samples had a chronic gastritis, and 20% of the patients had an intestinal metaplasia. Also, the prevalence of *cagA, iceA1,* and *iceA2* among their biopsies were reported as 87%, 58%, and 26%, respectively. However, we detected that 96% of our samples exhibited chronic active gastritis and 10 (40%) *H. pylori-positive* patients were positive for intestinal metaplasia as precancerous lesion. In addition, the prevalence of *cagA, iceA1,* and *iceA2* virulence genes in our *H. pylori*-positive samples were 72%, 48%, and 52%. Similar to the study carried out by Akar et al., we detected no significant correlation between the intestinal metaplasia and the prevalence of virulence genes, too. A similar study conducted in Saudi Arabia (neighboring Iran) exhibited that from 30 *H. pylori*-positive biopsies, 3 (10%) samples were *iceA1*- and *iceA2*-positive, while all three patients had gastric cancer (Bibi et al. 2017). Also, 11 (36.6%) biopsies in their research were *cagA*-positive, from which 8 patients belonged to the cancer group, and the *vacA* gene was detected in 28 (93.3%) biopsies (Bibi et al. 2017).

In 2012, Jung et al. (2012) investigated the role of the *dupA* gene cluster as a pathogenic gene pool of *H. pylori,* associated with gastrointestinal diseases. Studies in this group showed that there was no relation between the *dupA* gene and duodenal ulcer in patients; however, the results of a follow-up study exhibited that the complete cluster of *dupA* gene was associated with duodenal ulcer (Jung et al. 2012). This gene appears to be expressed by the type IV secretory system alongside the *vir* gene, and the total cluster of the *dupA* gene plays a significant role in duodenal ulcers. Therefore, a complete study of the genomic structure encoding pathogenic genes related to the gastrointestinal diseases is very important (Jung et al. 2012). The study conducted by Lu et al. (2005) in South Korea, Colombia, and Japan showed that only 6–12% of the specimens collected from patients with gastric cancer contained this gene. However, in our study, the prevalence of the *dupA* gene was 52% in patients with gastritis.

On the other hand, the *oipA* gene, as a *H. pylori* virulence determinant, encodes an extracellular inflammatory protein (Souod et al. 2015; Quiroga et al. 2005). Interesting findings were obtained by reviewing other studies in this regard and comparing them with the results of our research. The *oipA* gene is recorded with a frequency of 71.54% in the study of Souod et al. (2015), without any significant association with gastric injury. In another study by Queroga et al. (2005) in Spain, this gene was detected in 74% of their samples. Also, Dabiri et al. (2009) reported a prevalence of 55% for this gene in Iran. However, 56% of our specimens contained the *oipA* gene.

Due to the increased risk of gastrointestinal diseases in developed and developing countries, the study of causing organisms and the related virulence factors is necessary. In addition, considering the stomach cancer rates in 2020, Iran ranks 9th in the world, while considering the gastric cancer deaths, Iran ranks 6th (https://www.wcrf.org/cancer-trends/stomach-cancer-statistics/). The world ranking of Iran considering the rate of gastric cancer in women and deaths in these populations is 6th and 3th, respectively. Due to the significant role of *H. pylori* in gastric ulcer, duodenal ulcer, and gastric cancer, especially in developing countries, the increased prevalence of virulence genes, such as *cagA* and *vacA* is a problematic concern. According to our study, the frequency of all tested virulence genes was higher than 48%, while most patients had chronic gastritis. However, prolong chronic gastritis can cause a higher chance for the emergence of gastric cancers. Then, we need to use an appropriate treatment program for these patients to eradicate the bacteria or use sufficient protocols to prevent the infection by inhibiting the bacterial adhesion.

## Limitations

One of the most important limitations in this study was the small sample size for a prevalence study.

## Data Availability

All data generated or analyzed during this study are included in this published article.

## Abbreviations

CagA: Cytotoxin-associated gene A
vacA: Vacuolating cytotoxin A
dupA: Duodenal ulcer promoting gene A
iceA: Induced by contact with epithelium gene A
oipA: Outer inflammatory protein A
DNA: Deoxyribonucleic acid
PCR: Polymerase chain reaction
WHO: World Health Organization
BabA: Blood group antigen binding adhesin A
BabB: Blood group antigen binding adhesin B
SabA: Sialic acid-binding adhesin A
PAI: Pathogenicity island
RUT: Rapid urease test
